# Advancing Source Tracking: Systematic Review and Source-Specific Genome Database Curation of Fecally Shed Prokaryotes

**DOI:** 10.1021/acs.estlett.4c00233

**Published:** 2024-08-07

**Authors:** Blake G. Lindner, Rakin A. Choudhury, Princess Pinamang, Lilia Bingham, Isabelle D’Amico, Janet K. Hatt, Konstantinos T. Konstantinidis, Katherine E. Graham

**Affiliations:** † School of Civil and Environmental Engineering, Georgia Institute of Technology, Atlanta, Georgia 30332, United States; ‡ School of Biological Sciences, Georgia Institute of Technology, Atlanta, Georgia 30332, United States

**Keywords:** microbial source tracking, fecal contamination, bioinformatics, genomics

## Abstract

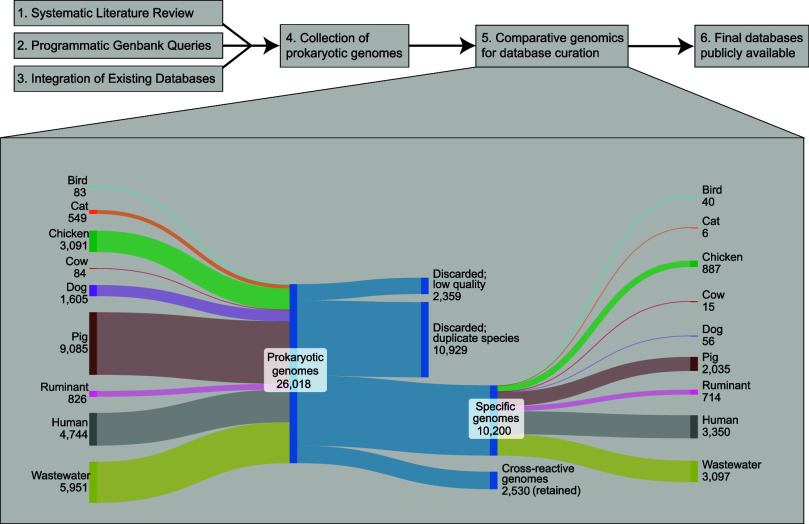

Advancements within fecal source tracking (FST) studies are complicated by a lack of knowledge regarding the genetic content and distribution of fecally shed microbial populations. To address this gap, we performed a systematic literature review and curated a large collection of genomes (n = 26,018) representing fecally shed prokaryotic species across broad and narrow source categories commonly implicated in FST studies of recreational waters (i.e., cats, dogs, cows, seagulls, chickens, pigs, birds, ruminants, human feces, and wastewater). We find that across these sources the total number of prokaryotic genomes recovered from materials meeting our initial inclusion criteria varied substantially across fecal sources: from none in seagulls to 9,085 in pigs. We examined genome sequences recovered from these metagenomic and isolation-based studies extensively via comparative genomic approaches to characterize trends across source categories and produce a finalized genome database for each source category which is available online (n = 12,730). On average, 81% of the genomes representing species-level populations occur only within a single source. Using fecal slurries to test the performance of each source database, we report read capture rates that vary with fecal source alpha diversity and database size. We expect this resource to be useful to FST-related objectives, One Health research, and sanitation efforts globally.

## Introduction

More than 120 million yearly cases of gastrointestinal disease result from exposure to sewage-impacted surface waters used for recreation.^1^ Health risks due to sewage contamination in the environment are assessed using fecal indicator bacteria (FIB).^2^ However, studies have shown that FIB can be insufficient for assessing aquatic fecal pollution, as they are nonconservative and nonspecific.^3−5^ These issues have born the field of microbial or fecal source tracking (MST or FST), which seeks to identify sources of fecal pollution in the environment and guide remediation.^6,7^

Historically, FST methods have implemented (q)PCR-based approaches to quantify source-specific genetic markers in samples from impaired waters to infer the presence of sewage. Genetic markers within the 16S rRNA gene of *Phoecaeicola dorei* (formerly *Bacteroides dorei*), such as HF183,^8,9^ or viral genetic markers, such as for crAssphage, have been used with success to identify sewage in the environment.^10^ However, this approach can suffer from low throughput.^11,12^ Higher throughput 16S rRNA gene sequencing has emerged as a potentially useful tool in the FST toolbox, with mixed results to date.^13,14^ Better characterization of source-specific fecally shed prokaryotic populations can assist with identifying genetic signatures that can more robustly attribute fecal sources in impaired waters.

Advancements in metagenomics have allowed for the characterization of prokaryotic genomes across many different microbiomes, especially the human gastrointestinal system. Datasets representing comprehensive efforts to collect species-level genomic representations of gastrointestinal prokaryotes are growing,^15−17^ covering an increasing number of animals. Yet, no database exists that collates this emerging data into a single repository for FST research. Leveraging publicly available data from isolation-based and metagenomic studies can reveal genomes and gene content shared between sources of fecal contamination thus helping identify source-specific features that could drive future innovation among FST methodologies.^18^

To this end, this systematic review and meta-analysis aimed to gather previously published prokaryotic genomes from studies of extraintestinal fecal shedding from the following animal sources: cow, pig, chicken, cat, dog, goat, seagulls, birds (non-seagull, non-chicken), and ruminants (non-cow). We aimed to assess two key questions: What prokaryotic genomes are specific to each source? How well do these collections of genomes capture fecal signal? To answer the latter, we collected, and shotgun sequenced fecal samples from several animal and wastewater sources in Georgia, USA, to test the read mapping performance of our databases. These efforts can be informative to practitioners of FST and those interested in developing new FST genetic marker assays based on the genomes of fecally shed prokaryotic populations.

## Methods and Materials

### Systematic Literature Review and Genome Collection

We systematically reviewed literature to collect prokaryotic genomes from existing studies on the fecal microbiomes of the eight animal source categories. PRISMA guidelines were followed during the systematic review.^19^ Search strings and detailed review protocols are documented in the SI.

Additionally, we sought to gather genomes into our collection by programmatically searching GenBank independently of searching published literature. Finally, we collected large genome databases stored elsewhere (e.g., Zenodo, EMBL, CNDB) from noteworthy publications or major studies produced after or missed by our initial literature review (particularly for human and wastewater sources).^20−23^ Further information on these methods can be found in the SI.

All genomes gathered through the above methods are recorded in the Supporting Information with relevant metadata and download sources for reproducibility (Tables S1, S2, and S3).

### Comparative Genomics and Database Curation

We constructed a software pipeline to process the genomes collected through our efforts. Documentation, scripts, and installation instructions are hosted online (https://github.com/blindner6/SourceApp). Additionally, a detailed summary of our workflow can be found in the SI. In brief, we used comparative genomic approaches to identify and discard genomes of poor quality, organize remaining genomes into species-level clusters (≥95% ANI), and flag (but retain) genomes belonging to different sources but assigned to the same species-level cluster as “cross-reactive”. In this way, all genomes contained in the finalized version of the database could be listed as source specific or cross-reactive (Table S4).

Our database is available for download online (10.5281/zenodo.10728776).

### Fecal Slurry Creation, DNA Extraction, and Illumina Sequencing

To characterize the performance of our genome databases, we collected fecal samples from animals that may contribute to surface water contamination in Atlanta, GA, USA. Fecal sources included: cow and pig (from one farm), dog and cat (from one shelter), as well as chicken and goat (from one farm). We also collected 1 L of 24-h-composited primary influent samples from three water reclamation facilities (WRF), and 500 mL of composited septage from a septage pumping truck. We created fecal slurries by homogenizing fecal samples from ten individuals of each source (dog, cat, chicken, goat, pig, cow) from which we extracted DNA and shotgun sequenced approximately 4.5 × 10^9^ bp (Gbp) for each. With equal amounts of sequencing effort applied to each slurry, we assessed the fraction of sequence diversity (an alpha diversity approximation) observed by the given sequencing effort via Nonpareil.^24^ Controlling our experiments in this way resulted in estimations for the coverage of a slurry’s sequence diversity that varied based only on community complexity (i.e., sequence diversity; Tables S5 and S6).

Additional details on the creation, sequencing, and analysis of these samples can be found in the SI. Short reads associated with the fecal slurries were deposited to NCBI under BioProject PRJNA1092107.

## Results and Discussion

### Systematic Literature Review and Data Collection

The systematic literature review revealed global trends to characterize animal fecal prokaryotes at the whole-genome level. Several sources had few numbers of studies producing prokaryotic genomes from feces, such as seagulls (0 studies) and cats (n = 1 study). Birds (ducks, starlings, geese, finches, starlings, wigeons, swallows, crows, pheasants), cows, pigs, and ruminants, each had more than 10 studies (Table S1). The systematic literature review also revealed many different sequencing and bioinformatic methods used for data processing and genome binning. Culture-based methods were the most common. Some studies used shotgun metagenomes (a culture-independent approach) to recover metagenome-assembled genomes (MAGs), but this approach was less common. Most studies used short-read platforms (e.g., Illumina) to generate sequencing data, but some used long-read platforms (e.g., PacBio) either individually or as a hybrid sequencing approach.

### Curation and Comparative Genomics of Source Databases

**Figure 1:**
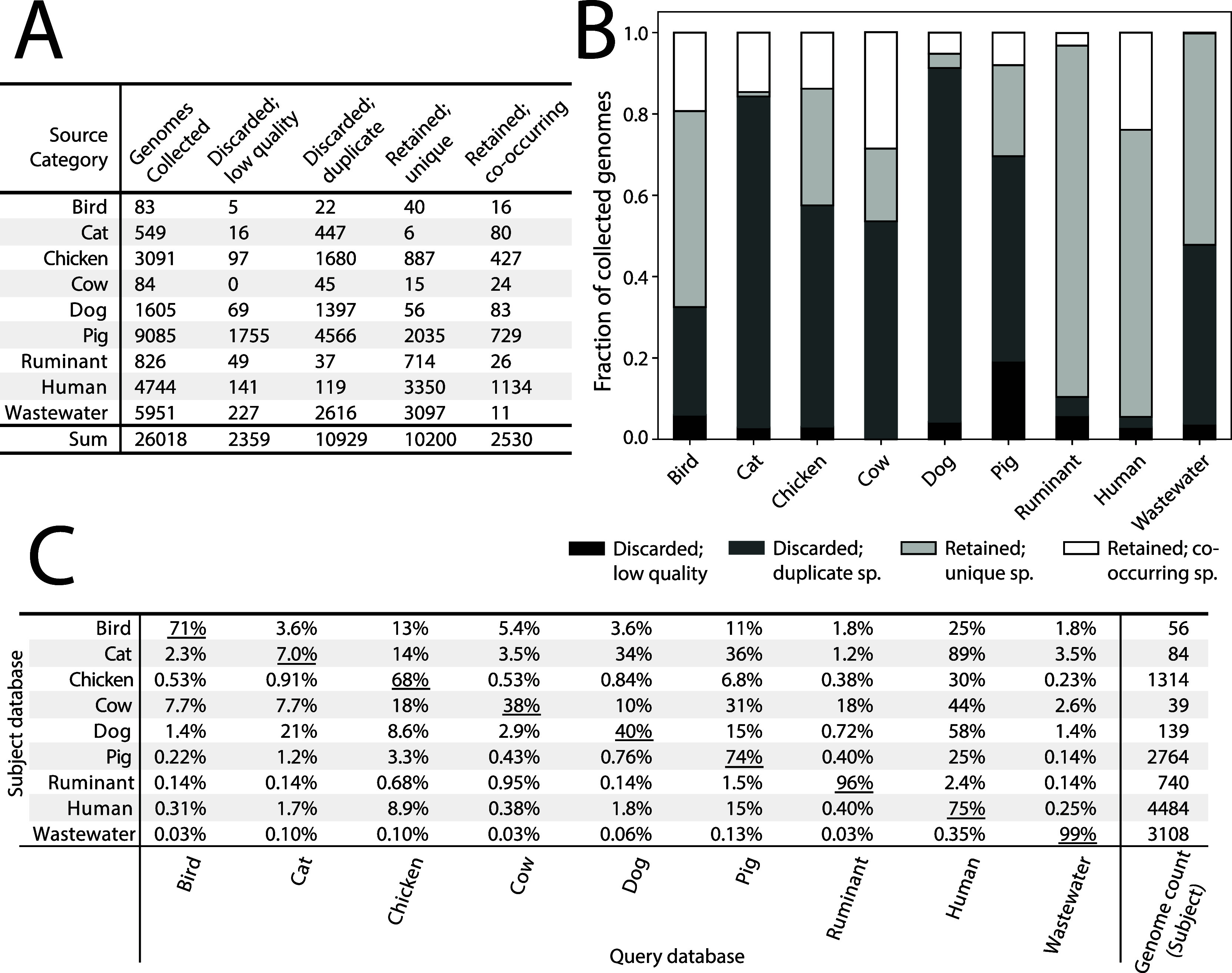
Overview of genome search results and curation of source databases. (A, B) The total genomes found for each source and their outcome in the study. (C) Matrix summary of the fraction of genomes representing shared species across source categories as revealed by querying all versus all genomes from the databases in search of species-level matches (i.e., ≥95% ANI). Underlined values on the diagonal denote the fraction of source-specific genomes for a given source database (i.e., the fraction of genomes within a source database with no external species-level matches). Definitions: “Discarded; low quality” refers to genomes recovered from literature review but rejected due to having qualities below the selected quality threshold (Q < 50). “Discarded; duplicate sp.” refers to genomes removed from the source database due to overlap with another greater quality genome in the same source database representing the same species. “Retained; unique sp.” refers to genomes retained in the source database with no species-level matches among the other source databases. “Retained; co-occurring sp.” refers to genomes retained in the source database which matched other genome(s) within another source database(s) at the species-level.

We collected 26,018 genome sequences representing fecally shed prokaryotic populations across the selected sources. Figure 1 summarizes the number of genomes collected for each source, their outcomes following our examination, and to what extent genomes were unique to a source. Following our curation efforts, 2,359 genomes were discarded due to poor quality scores. An additional 10,929 genomes were discarded during species-level clustering (≥95% ANI^25^), which aims to leave only a single representative genome for each species-level cluster within each source. Thus, the remaining 12,730 genomes represent species found in each of our sources. Of these genomes, 2,530 (19%) represent species observed in multiple sources (i.e., cross-reactive species; Figure 1C).

The fraction of species in the curated database specific to a given source varied substantially. For example, the human genome collection contained the highest number of cross-reactive prokaryotic species (25% or 1,134) but was also the collection with the greatest number of species represented altogether (n = 4,484 species). The much smaller cat and dog collections exhibited more cross-reactivity, with 93% of cat-associated species (n = 77) and 60% of dog-associated species (n = 81) co-occurring in other sources. In this instance, nearly all the cross-reactive cat and dog species were also found in the human database. In contrast, the wastewater collection exhibited virtually no cross-reactivity with any other source (11 species; 0.4%). In general, the largest numbers of shared species were between either (1) the bird, cat, chicken, cow, dog, and pig databases with the human data, or (2) between source categories that are closely related in habitats (e.g., dogs and cats).

**Figure 2:**
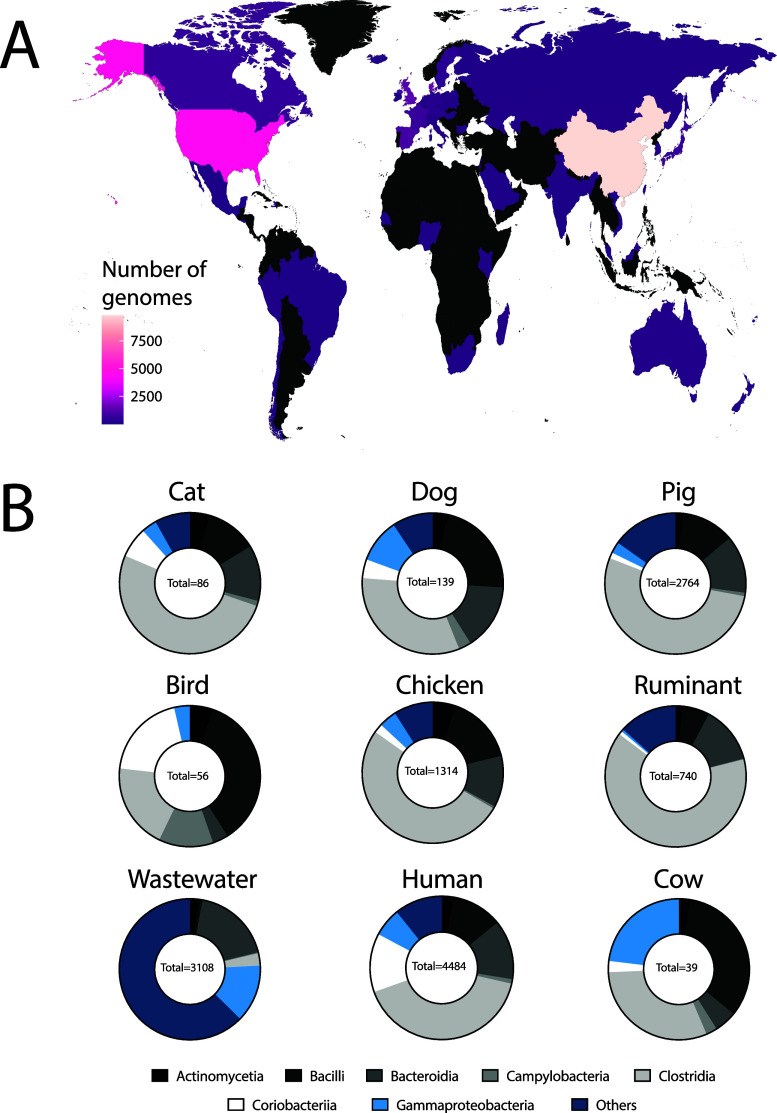
(A) Geographic distribution of genomes (quality scores ≥50) and (B) number of species within a taxonomic Class accessioned within the MST genome database (*n* = 12,730 genomes). The top seven Classes are shown while remaining Classes are grouped together under “Others”.

We summarized the location metadata associated with all genomes collected (regardless of their inclusion in the finalized source databases) to show the geographic breadth of the data collected (Figure 2A). Most genomes originated from China (n = 9,780) and the USA (n = 4,274), though many genomes (n = 3,674) had no metadata describing their geographic origin. It is crucial to note that our efforts returned comparatively few genomes from countries in the Southern Hemisphere.

We examined common and unique taxonomies across source categories, as we dereplicated genomes within source databases at the species level (Table S4). Gut-associated classes, such as *Actinomycetia*, *Gammaproteobacteria*, *Bacilli*, *Clostridia, Campylobacteria, and Bacteroidia*, were common across all source categories. The larger genome databases also had unique taxonomies (e.g., human, wastewater), likely owing to their more complete characterization relative to the underexplored sources (e.g., cow). For instance, *Desulfitobacteriia, Halobacteria,* and *Thermoanaerobacteria*, were uniquely found in the human fecal genome database. The wastewater database had 157 unique Classes compared to the other source category databases.

### Assessing Database Performance

**Figure 3:**
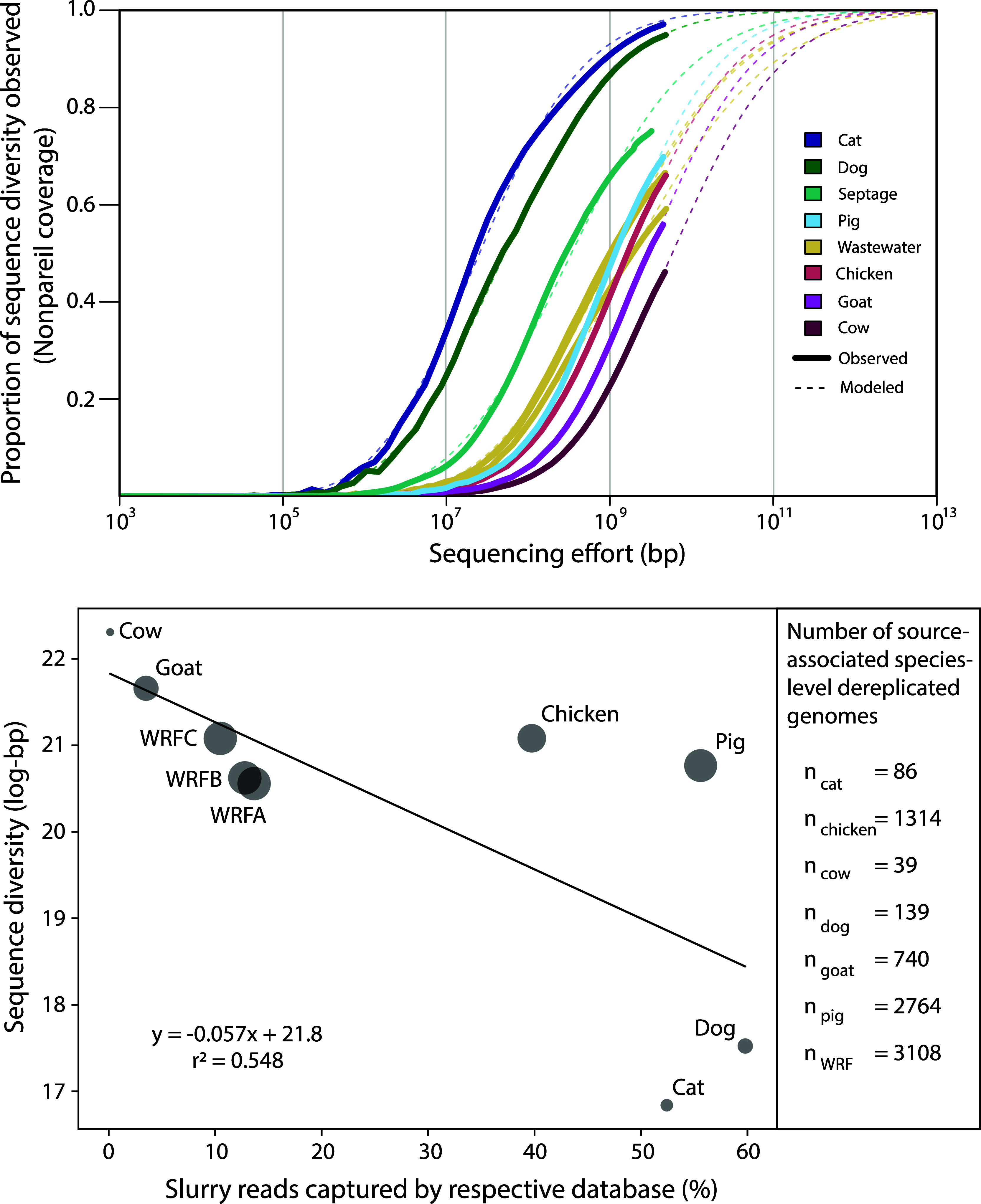
Fecal slurry sequencing results and genome database performance. (Top) The fraction of sequence diversity observed (*y*-axis) for each fecal slurry as estimated by Nonpareil shown as functions of sequencing effort (*x*-axis). The underlying data provided to Nonpareil were short read metagenomes of fecal slurries from source categories used in this study. Solid lines represent the observed relationship and dotted lines represent extrapolated models; approximately 4.5 Gbp of sequencing effort per sample was used. (Bottom) Nonpareil diversity for each slurry as a function of reads mapped from the slurry to its respective curated genome database. The size of each point is scaled to represent the number of species-level dereplicated genomes represented in the respective source database, and the size scaling value was determined as (1 + ln(n/n_cow_)) × 100. The number of species-level dereplicated genomes present in each source database are shown for reference in the bottom right panel.

Laboratory-assembled fecal slurries, primary influent, and septage were filtered, extracted, sequenced, and the diversities of their resulting metagenomes compared across sources. Values for the percentage of observed sequence diversity (or Nonpareil coverage^24^) ranged from 46% (cow) to 97% (cat) (Figure 3, Top; Table S5). This reveals that for more complex slurries (e.g., cow), substantially greater amounts of sequencing effort are required to cover most of the community’s diversity. For example, approximately 1–4 Gbp of sequencing effort would be required to observe 95% of the sequence diversity for the least diverse fecal slurries (cat) whereas approximately 400 Gbp would be required to achieve that level for the most diverse fecal slurry (cow). These results can inform the planning of studies utilizing assembly and binning of shotgun metagenomic data to recover new genomes for a particular source category.^26^

Next, to understand the representativeness of each source database, we mapped each slurry’s short reads to their respective genome databases (i.e., dog fecal slurry short reads queried against the dog database). Although we expect these databases to continue growing alongside increasing efforts to catalog prokaryotic diversity, this type of benchmarking can elucidate which databases are robust in covering the source’s genome diversity. We assessed what fraction of the short-read data each database could capture, with the expectation that higher rates of read capture imply a more representative database. When plotting sequence diversity as a function of slurry read capture by their respective databases, a linear relationship was found (Figure 3, bottom). The dog and cat databases underperformed relative to this trend whereas both chicken and pig databases overperformed, though these databases vary substantially in size (Figure 2). Thus, additional variables may be crucial for understanding these trends such as the number of source-associated species in each database and their abundance rank. As each source’s genome database becomes increasingly speciose, not only should read capture rates increase, but also the utility of this resource for informing developments in FST. Lastly, future work is needed to contextualize the performance of these databases for use in metagenomic FST with field testing or mesocosm experiments. Such efforts may require development of similar genome database(s) for environmentally associated (e.g., marine, freshwater) prokaryotic genomes.

**Figure 4:**
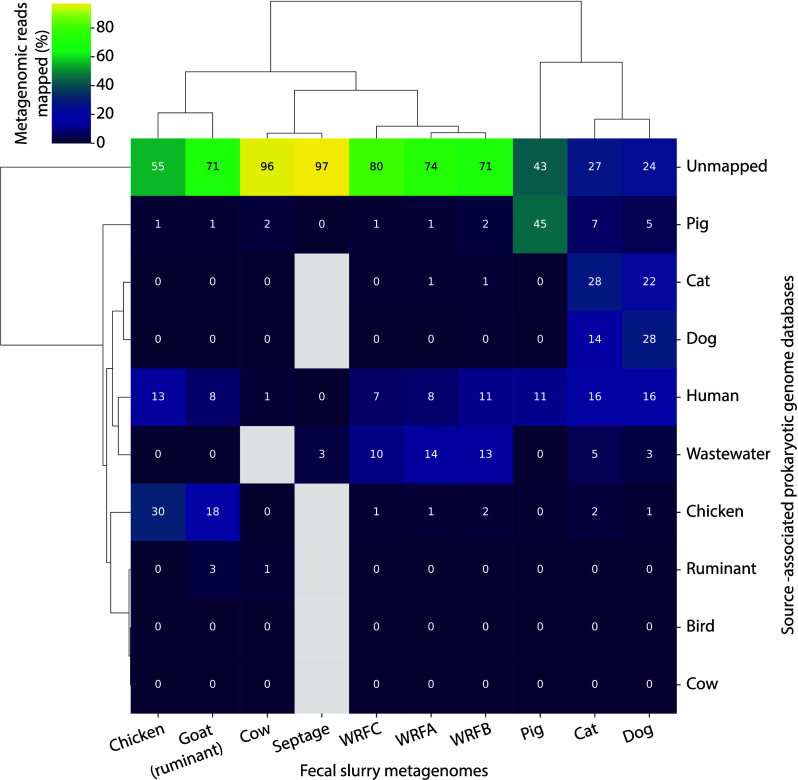
Competitive short read mapping performance of metagenomic reads from experimental slurries (columns) prepared in the laboratory mapped to our curated genome databases (rows). Reads were competitively mapped to genomes within databases and summed within each database to obtain the percentage of reads mapped, which are denoted by the values shown. Values are rounded to integers and gray indices without a value are true zeroes. Both rows and columns have been clustered to minimize variance within clades using Ward’s method. WRF = wastewater reclamation facility.

To understand the degree to which these source databases manage instances of cross-reactivity, we competitively mapped each fecal slurry metagenome against every source database to obtain new read capture rates (Figure 4; Table S6). Metagenomic read captures rates vary across studies due to many factors but are typically improved by the inclusion of MAGs recovered from the samples themselves. These results did not include assembly and binning of the fecal slurry metagenomes, as the goal was to test the efficacy of each source database without the addition of *de novo* references (i.e., new MAGs). For example, the UHGG catalog of human-associated prokaryotic genomes (n = 204,938 genomes; 4,644 species) typically recovered between approximately 65–85% of metagenomic reads.^20^ In contrast, our total recovery rates varied from approximately 75% (dog) to 5% (cow) as seen in Figure 4 following the size of the source database and complexity of the fecal slurry. These values were slightly lower compared to the noncompetitive exercise discussed above (Figure 3, bottom).

For most fecal slurry metagenomes, its respective genome database was the dominant database against which reads were mapped, except for the cow and goat slurries. We expected the goat slurry to map predominantly to the ruminant database, however, it best mapped to the chicken database. Other instances of cross-reactivity from fecal metagenomes to nonmatching genome databases were observed, especially for cat and dog metagenomes, as they frequently mapped to each other and to the human database. The effect of shared facilities for some of the sources may have contributed to the overlap we observed in mapped reads among these sources (e.g., dog and cat, chicken and goat). This is a key limitation for using source-associated genome databases in this context which will have to be addressed by future efforts developing shotgun metagenomic FST.

### Relevance for Environmental Monitoring

Efforts metabarcoding the 16S rRNA gene previously revealed degrees of source specificity for the microbiota shed in animal feces (and wastewater) which have proven transformative for FST practice.^27−32^ Indeed, many of the most widely used FST genetic markers target the 16S rRNA gene and their development was precipitated by inquiry into the distribution of 16S sequences across various sources.^6^ However, 16S rRNA gene sequences are not robust species-level delineators.^14^ Thus, the associations of prokaryotic species within fecal sources have not been thoroughly investigated but could be similarly useful in driving the next generation of FST advancements. In part, this research gap exists because species-level reference genomes have yet to be synthesized into databases dedicated to FST applications. We suggest that the construction (and ongoing maintenance) of source-associated whole genome databases is a practical step toward such innovation–including metagenomic based FST – and is therefore a needed resource for the community. This study provides the most comprehensive genomic databases to date cataloging fecally shed prokaryotes from commonly implicated sources responsible for surface water impairment. We have automated the procedure for constructing these databases which will facilitate future expansion as more genome sequences become available. Additionally, our fecal slurry metagenome read mapping exercises represent a straightforward approach to assess the representativeness of each source database.

Overall, we observed relatively low levels of species cross-reactivity across most source categories (Figure 1). Yet, our results have shown that these databases are far from representative for several sources, contributing to cross-reactivity in the metagenomic read mapping exercises due to species (or gene content) which span source categories, but remain unaccounted for in the databases themselves. (Figure 4). Therefore, updating our views on this topic will be essential as additional genomes are described for fecally shed prokaryotes from understudied sources and geographies (Figure 2). Despite our currently incomplete views, low cross-reactivity of species between sources is plausible and consistent with other work suggesting many of the symbiotes inhabiting fecal microbiomes could be the products of long-term coevolution with their host while also influenced by other factors such as host diet and geography.^27,33−35^ Indeed, even the largest source databases, in terms of species count, exhibited overlaps of less than or equal to 30% of observed species (e.g., humans, pigs, and chickens; Figure 1).

Our analysis also revealed strikingly low cross-reactivity between wastewater and animal sources, including humans (i.e., 1% of wastewater species were shared among other sources). What then explains the uniqueness we report for the wastewater genome database, and is it a function of the incompleteness of the available genomic data? Others have reported sewer infrastructure to harbor unique reservoirs of microbial diversity which proliferate and outcompete most human-associated taxa within wastewater collection and conveyance–sometimes referred to as “microbial weeds”.^18,36,37^ We see the preponderance of unique species in the wastewater database as indicative of those taxa composing the microbiome associated with wastewater collection and treatment processes. Indeed, the studies from which we recovered genomes described from wastewater included samples taken from raw or primary influent and biological processes (e.g., activated sludge).^21−23^ In this way, the wastewater genome database alone can serve as a resource for studies of wastewater biological processes as well.

The human gut microbiome represents the best studied fecal source with work summarizing our understanding of its extant species diversity estimating it is probably comprised of about 3,000 reoccurring (i.e., excluding singletons) species globally.^20^ Surveys of human feces at the metagenomic level previously reported sequence diversity values less than those we found for our wastewater samples but greater than those of the dog and cat fecal slurries.^38,39^ Unsurprisingly, this suggests there are a substantial number of species missing from the databases of many of the sources we examined. Indeed, no source saw read capture rates exceed 60% in our noncompetitive read mapping exercises (Figure 3). Considering the risk implications of cow-derived fecal contamination in recreational waters and the relatively few publicly available genomes from cow feces (n = 84), our work highlights the need for more substantial research efforts to understand microbial communities in cow feces as opposed to the rumen alone (Figure 1).^40^ Our work also highlights the need for more global surveys of fecally shed prokaryotes to address issues of variation in fecal diversity among individuals of the same source (e.g., biodiversity; Figure 2).

Several bioinformatic tools exist that utilize sequencing data for source tracking, including FEAST, FORENSIC, and (meta)SourceTracker for which our database may be useful in efforts to construct OTU tables.^29,41,42^ Each of these tools uses statistical models to estimate the inputs of sources to sinks. As such, sources must be provided with sink data sets to characterize the attributions of sources to each sink sample, which are not always readily available when working on issues of surface water contamination from point or nonpoint sources. Though, in their current state, these source-specific databases are far from complete, owing to the relatively limited information we have on animal fecal microbiomes; therefore, we caution against drawing broad biological or ecological conclusions from our summaries above. For instance, there were no publicly available assemblies from the feces of seagulls, although they are often a source of pollution to surface waters and can carry human pathogens.^43−45^ This work highlights the need for ongoing research on animal fecal microbiomes across many species, geography, and lifestyles, as well as septage to inform FST efforts and One Health research.

## Supporting Information Available

Additional methods for systematic literature review, database construction, and comparative genomics (PDF)
Summaries of systematic literature review results, genome collection, and results of comparative genomic analyses: Tables S1–S6 (XLSX)
